# Point-of-care ultrasound in the evaluation of STEMI patients

**DOI:** 10.3389/fcvm.2025.1627396

**Published:** 2025-09-02

**Authors:** Marco Vugman Wainstein, Guilherme Pinheiro Machado, Guilherme Heiden Telo, Anderson Donelli da Silveira, Luiz Antônio Nasi, Gustavo Neves de Araujo

**Affiliations:** ^1^ Cardiology Department, Hospital de Clínicas de Porto Alegre, Porto Alegre, Brazil; ^2^ Cardiology Department, Hospital Unimed Grande Florianópolis, Florianópolis, Brazil

**Keywords:** STEMI, point-of-care ultrasound, lung ultrasound, LVOT-VTI, acute heart failure, cardiogenic shock

## Abstract

**Background:**

ST-elevation myocardial infarction (STEMI) remains a leading cause of morbidity and mortality globally. Early risk stratification and detection of complications are critical for optimizing patient outcomes. Point-of-care ultrasound (POCUS) has emerged as a valuable bedside tool in the acute evaluation of STEMI patients.

**Objectives:**

To explore the role of POCUS in the early assessment of STEMI patients, focusing on its diagnostic and prognostic utility.

**Methods:**

A comprehensive review of current literature was conducted, examining the application of POCUS in STEMI.

**Results:**

Lung ultrasound (LUS) enables rapid detection of pulmonary congestion through the identification of B-lines, offering superior sensitivity compared to traditional methods. Left ventricular outflow tract velocity-time integral (LVOT-VTI) provides a quantitative assessment of stroke volume and cardiac output, aiding in the identification of low-flow states. Integrating these modalities enhances hemodynamic evaluation. Moreover, a systematic POCUS assessment may facilitate early detection of high-risk patients with acute heart failure or cardiogenic shock, as well as the identification of mechanical complications.

**Conclusions:**

The incorporation of POCUS, specifically LUS and LVOT-VTI, into the early evaluation of STEMI patients enhances diagnostic accuracy and prognostic assessment. Future research should focus on standardizing protocols and evaluating the impact of POCUS-guided management on patient outcomes.

## Introduction

ST-elevation myocardial infarction (STEMI) is associated with significant morbidity and mortality. Despite significant advances in pharmacological and reperfusion strategies, particularly percutaneous coronary intervention (PCI), early risk stratification and adequate in-hospital management remain pivotal in optimizing outcomes (1). Timely identification of high-risk features and associated complications such as acute heart failure (AHF) or cardiogenic shock (CS) can guide appropriate clinical management, from intensive monitoring to urgent mechanical support.

Conventional evaluation in myocardial infarction relies on clinical examination, electrocardiography, and biomarkers. However, these tools have limitations, particularly in detecting early hemodynamic deterioration or guiding fluid and pharmacologic therapy. For example, the cornerstone Killip classification (2) is a useful tool, however, it may sometimes be imprecise. The distinction between Killip groups II and III is defined by the level on the chest at which rales are audible, making it difficult to distinguish, especially in a noisy environment such as an emergency room (2). As the complexity of STEMI patients increases—with increasing age and prevalence of comorbidities such as diabetes, chronic kidney disease, and prior heart failure—there is a growing need for dynamic, bedside tools that can provide real-time insights into cardiac function, volume status, and pulmonary congestion.

Point-of-care ultrasound (POCUS) has emerged as a rapid, non-invasive assessment of cardiac and pulmonary parameters, and has been suggested as a fifth pillar of bedside physical examination (3). Its portability, user-friendliness and diagnostic accuracy have contributed to its broad integration into emergency medicine, critical care and cardiology. In the setting of STEMI, POCUS can play a pivotal role as an early triage and prognostic modality, facilitating the identification of acute complications, alternative diagnosis, and guiding hemodynamic optimization.

**Figure 1:**
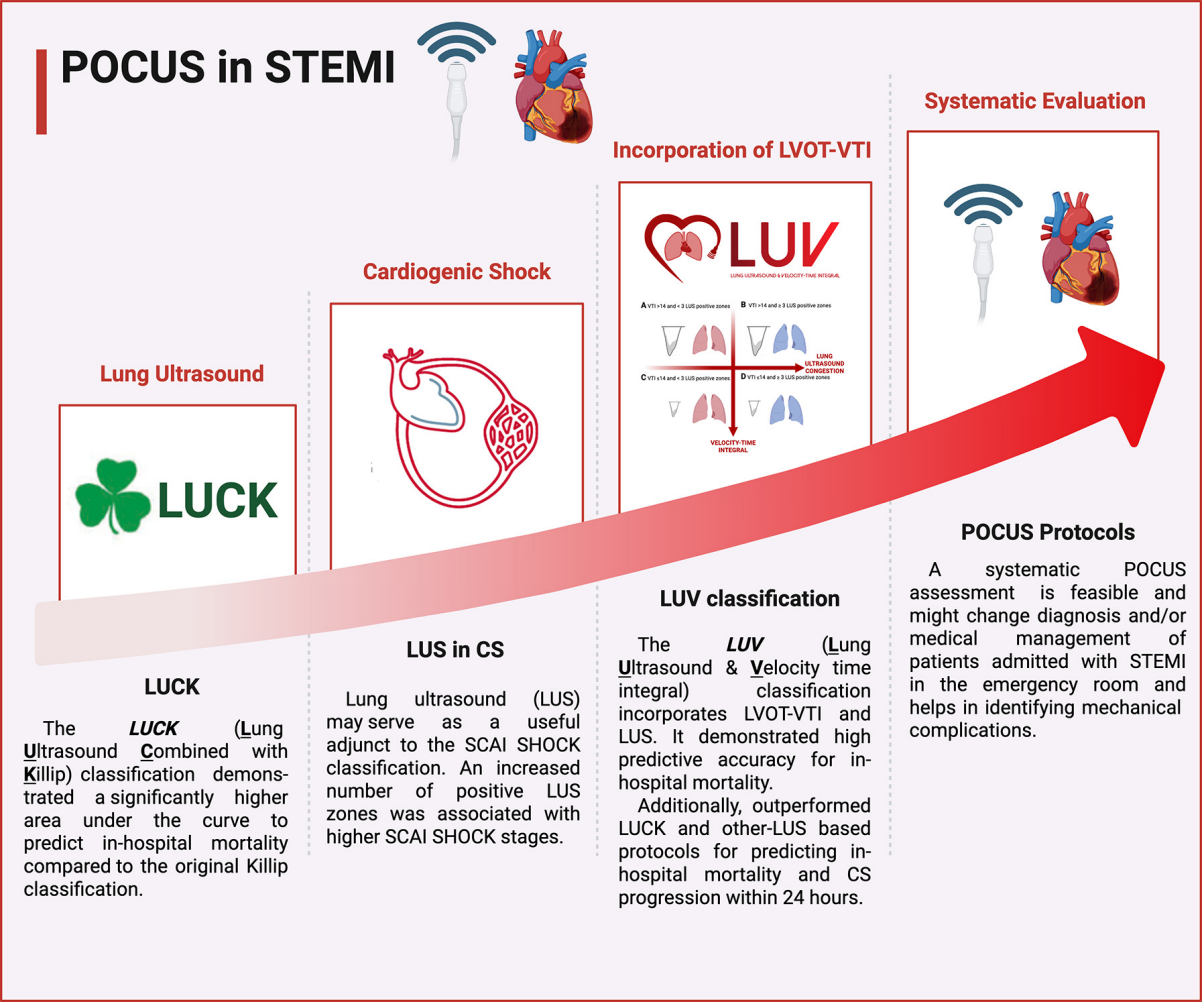
Central Illustration. Evolution of point-of-care assessment in ST-elevation myocardial infarction. Figure created in Biorender.com.

This manuscript explores the evolving role of POCUS in the early evaluation of STEMI patients, with a particular emphasis on lung ultrasound (LUS) and the measurement of left ventricular outflow tract velocity-time integral (LVOT-VTI). By integrating current evidence and outlining future perspectives, this review aims to emphasize the clinical significance of POCUS in enhancing risk stratification and guiding early therapeutic decision-making in the management of STEMI (Figure 1—Central Illustration).

## Point-of-care ultrasound in contemporary practice

POCUS has improved bedside assessment across various medical specialties, particularly in acute and critical care settings. Once considered an adjunct to physical examination, POCUS is now recognized as a frontline diagnostic tool, offering real-time, reproducible insights into cardiopulmonary physiology, volume status, and organ perfusion (4–6).

In contemporary clinical practice, the limitations of the physical examination are well recognized. While auscultation and inspection remain fundamental, they are hampered by substantial interobserver variability and limited sensitivity in detecting subtle or evolving pathologies. For example, the sensitivity of rales to detect right atrial pressure ≥10 mmHg and left atrial pressure ≥20 mmHg are 28% and 25%, respectively (7). The sensitivity of jugular venous distension for identifying elevated right atrial pressures follows the same trend, being only 39% (8). In contrast, POCUS allows for direct visualization of underlying pathophysiological processes—such as B-lines on lung ultrasound indicative of pulmonary edema, a plethoric inferior vena cava (IVC) suggestive of elevated right atrial pressures, or a mechanical complication on focused cardiac ultrasound. A growing body of evidence demonstrates that incorporating POCUS into the physical examination markedly enhances diagnostic accuracy, particularly in patients presenting with undifferentiated dyspnea (9), shock (10) or chest pain (11). In patients admitted with acute dyspnea, for example, the lung ultrasound sensitivity to detect acute pulmonary edema is 97% (12).

In the emergency department, POCUS protocols such as the Focused Assessment with Sonography in Trauma (FAST) (13), the Rapid Ultrasound in Shock (RUSH) (14) exam, and the Bedside Lung Ultrasound in Emergency (BLUE) (12) protocol have been well validated. These approaches streamline diagnostic workflows, shorten the time to diagnosis, and often lead to immediate changes in management. Similarly, in the intensive care unit, POCUS is increasingly used for guiding fluid resuscitation, evaluating response to therapy, and identifying reversible causes of hemodynamic instability (14–16).

Cardiovascular applications of POCUS are expanding rapidly. Focused cardiac ultrasound (FoCUS) (17) can identify pericardial effusion, assess left ventricular systolic function, and evaluate valve dysfunction with high specificity. LUS and Venous Excess Ultrasound (VExUS) identify pulmonary and systemic congestion with excellent accuracy in acute heart failure, serving as useful tools for both diagnosis and decongestion management (18), as well as a potential application on acute coronary syndromes (19, 20) and transcatheter aortic valve implantation (21, 22).

Importantly, POCUS is not intended to replace comprehensive echocardiography or advanced imaging modalities. Rather, it serves as a dynamic, bedside extension of clinical evaluation—allowing for real-time reassessment and serial monitoring. As such, its utility is enhanced when performed by clinicians directly involved in patient care, who can integrate findings immediately into therapeutic decisions.

## Lung ultrasound

For many years after the introduction of ultrasound technology, it was commonly accepted that ultrasound imaging was not useful for the evaluation of pulmonary parenchyma, due to the poor transmission of ultrasound waves through air. It was not until the late 1990s that lung ultrasound was popularized, with pioneering applications in critically ill patients, including the detection of acute pulmonary edema (23). Since then, there has been a continuous and growing body of evidence supporting its clinical utility, particularly in cardiology (24).

Lung ultrasound provides a direct, real-time visualization of extravascular lung water through the detection of B-lines—vertical, hyperechoic artifacts arising from the pleural line and moving with respiration. These artifacts reflect increased fluid within the interlobular septa and alveolar interstitium, making them a sensitive marker of pulmonary congestion, defined as at least two positive sites (≥3 B-lines each) bilaterally (25). Studies have demonstrated that LUS is superior to both auscultation and chest radiography in detecting pulmonary edema, with diagnostic accuracies exceeding 90% in many acute care settings (26). It is important to emphasize that, although a single positive zone does not strictly define pulmonary congestion, its presence may still carry prognostic implications, as will be discussed below. Therefore, for this review, we will define “pulmonary congestion” as the presence of one or more lung zones demonstrating three or more B-lines.

Since its introduction and particularly over the last fifteen years, the cardiology community has increasingly recognized the potential of LUS to aid in the diagnosis and management of both acute and chronic heart failure (27). A notable example of the method's utility in this setting is a randomized trial demonstrating that tailored LUS-guided diuretic therapy for pulmonary congestion reduced the incidence of heart failure decompensations and improved walking capacity in ambulatory patients, compared with standard follow-up (28). Although LUS was first investigated in patients with acute coronary syndromes in 2010 (29), it was not until 2020 that it was specifically studied in patients with STEMI (30).

**Figure 2:**
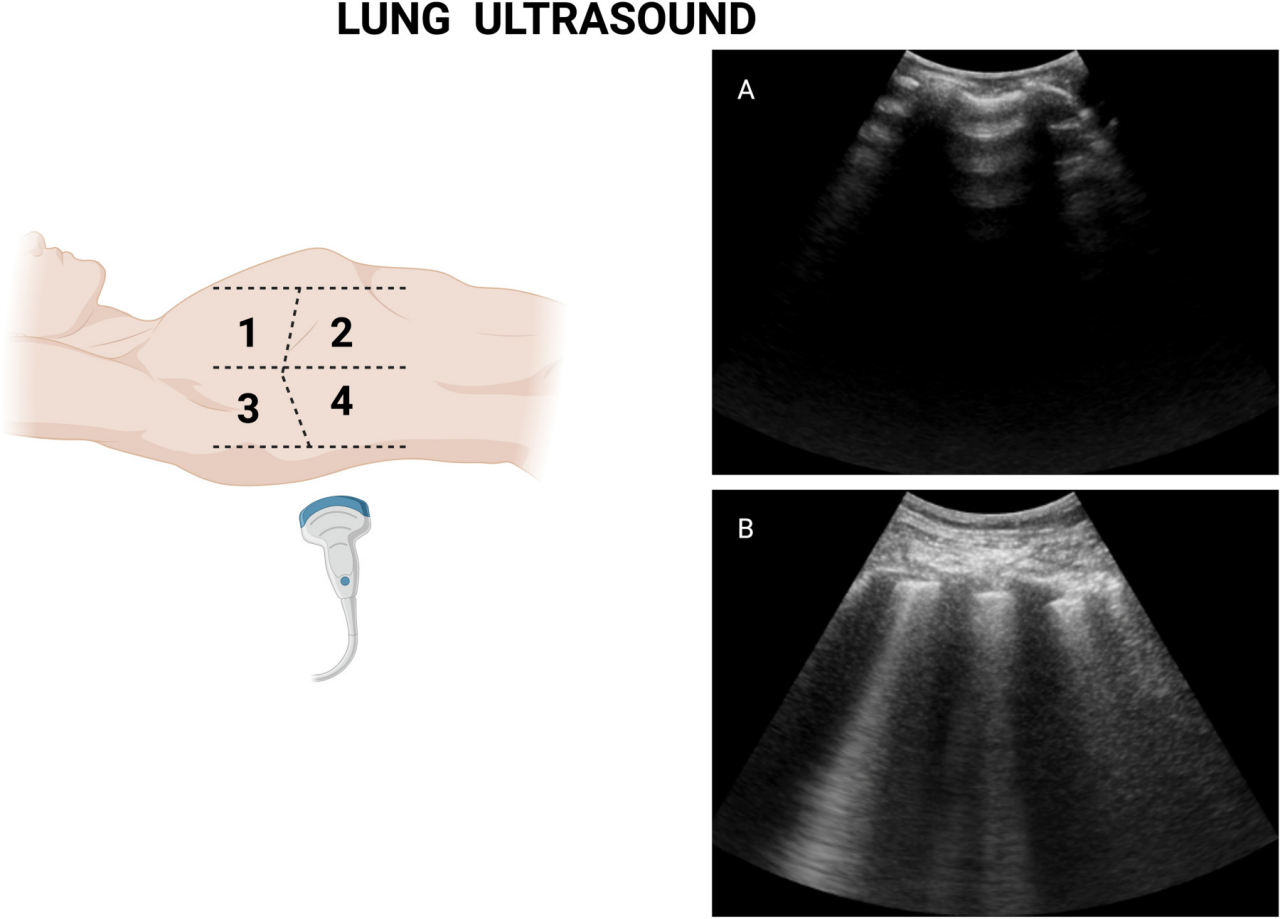
Lung ultrasound measurement illustration. **(A)** Normal lung with air-filled alveoli. **(B)** Congested lung with multiple B-lines (comet tail appearance). Figure created in Biorender.com.

In the context of STEMI, LUS can be performed rapidly at the bedside, typically within a few minutes using an 8-zone scanning protocol (25) (Figure 2). Importantly, the identification of B-lines does not require advanced ultrasound training, as interobserver agreement among trained clinicians is high (30, 31), and it is suggested that one morning of hands-on experience or even a standardized internet-based module of two hours is enough to achieve excellent reproducibility in the identification and quantification of B-lines (24). Of note, both curvilinear and phased array transducers may be recommended for LUS assessment, as there is a good correlation among users who had at least one month of POCUS training (32). The use of the sectorial transducer may be unfamiliar to non-cardiologists or doctors who are not as experienced with this type of transducer. However, an interesting analysis by Walsh (32) and colleagues compared both transducers for lung ultrasound assessment and found a good correlation among trained users.

## Left ventricular outflow tract velocity-time integral

**Figure 3:**
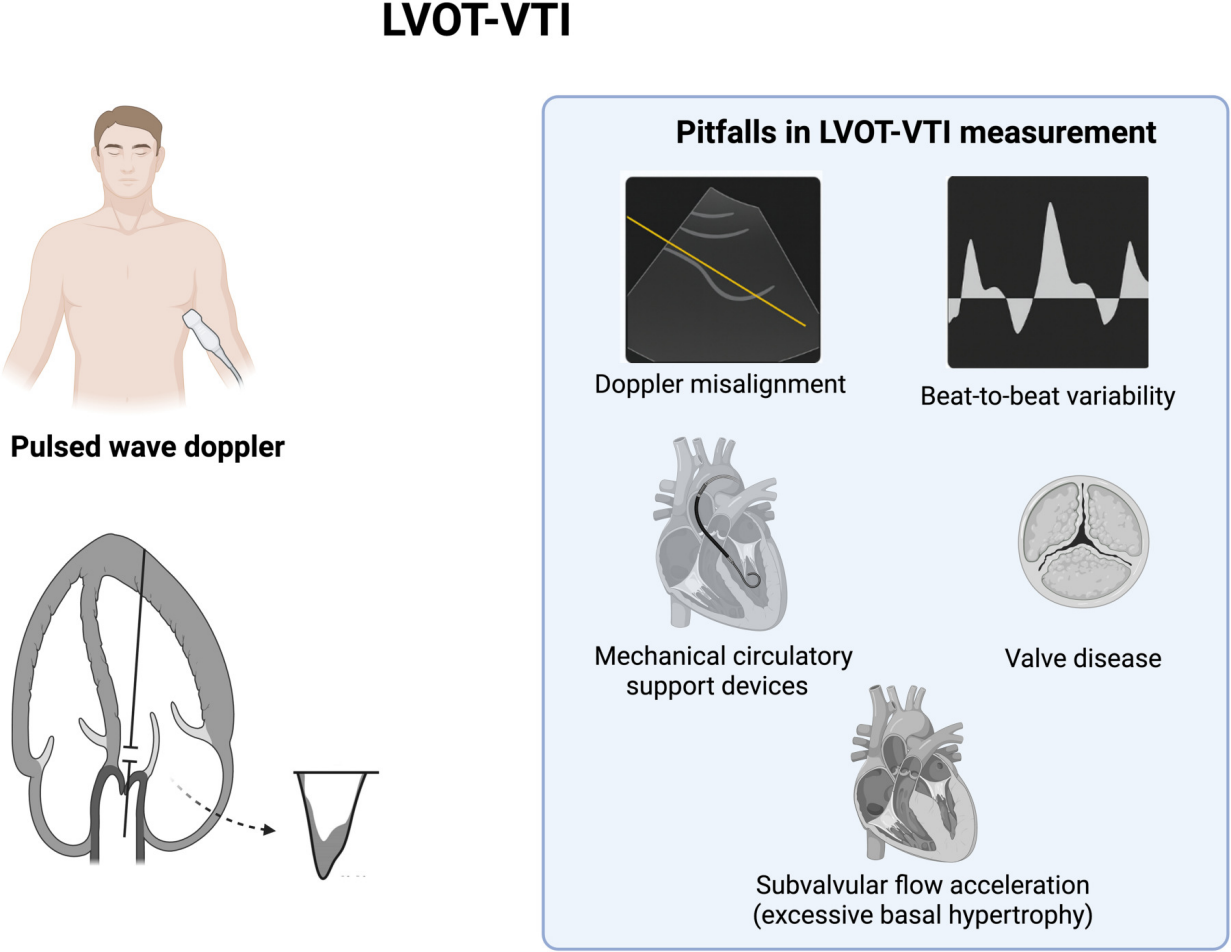
LVOT-VTI measurements illustrations and common pitfalls. Figure created in Biorender.com.

LVOT-VTI is a Doppler-based measurement that estimates stroke volume and, by extension, cardiac output. It is obtained via pulsed-wave Doppler at the level of the LV outflow tract in the apical five-chamber (Figure 3). The VTI represents the distance that blood travels during one cardiac cycle; when multiplied by the LVOT cross-sectional area and heart rate, it yields cardiac output. Five to seven cardiac cycles should be averaged for a patient in atrial fibrillation (33). In practice, however, VTI alone (without indexing to LVOT area) is often sufficient as a bedside surrogate of flow, with normal values typically ranging between 18 and 22 cm. Values ≤16 cm are considered indicative of an impaired forward flow, can provide insights into cardiac function even when the LVEF is preserved and it is a strong predictor of mortality in critically ill patients (34). Moreover, it can predict hemodynamic deterioration even when classic signs of cardiogenic shock are absent.

While LUS provides valuable insight into pulmonary congestion, it does not directly quantify cardiac output or systemic perfusion. In acute coronary syndromes—particularly STEMI complicated by heart failure or cardiogenic shock—congestion and hypoperfusion may coexist, and distinguishing between these hemodynamic states is critical for appropriate management. In this context, adding a measure of forward flow, such as the LVOT-VTI, to LUS protocols enhances both diagnostic and prognostic utility as will be discussed.

The LVOT-VTI is a valuable echocardiographic parameter for estimating stroke volume and cardiac output. However, its accuracy can be significantly affected by technical and physiological factors. Common pitfalls include poor Doppler beam alignment, misinterpretation of waveforms, and inadequate averaging techniques. Moreover, certain clinical conditions—particularly moderate-to-severe aortic regurgitation and subaortic obstruction (either fixed or dynamic)—can lead to substantial overestimation of VTI. Dynamic left ventricular outflow tract obstruction (LVOTO), for instance, may occur in settings of severe hypovolemia or in patients with asymmetric septal hypertrophy, especially under conditions of low preload and heightened inotropic stimulation. Both dynamic LVOTO and aortic regurgitation increase flow velocities through the outflow tract, leading to falsely elevated VTI values. Additionally, the presence of mechanical circulatory support devices such as Impella may interfere with native flow dynamics and Doppler signal interpretation, further complicating VTI assessment. When technical quality is suboptimal or when interfering pathology is present, reliance on serial trends or adjunctive hemodynamic parameters is advised (35). Despite its limitations, LVOT-VTI remains a practical and dependable tool for bedside assessment of hemodynamic status, especially when performed by trained physicians in emergency and critical care settings.

## Importance of prognostic evaluation in STEMI

The management of STEMI has evolved significantly over recent decades, primarily due to advances in reperfusion therapy and pharmacological management. Nevertheless, STEMI remains a major cause of morbidity and mortality worldwide. While early reperfusion is the cornerstone therapy, outcomes are substantially influenced by the patient's baseline risk profile and the development of complications such as acute heart failure, arrhythmias, or cardiogenic shock. In this context, accurate and timely prognostic evaluation is critical—not only to guide immediate therapeutic decisions but also to guide resource allocation, determine level of care, support early discharge, and anticipate long-term outcomes.

Traditional prognostic tools such as the TIMI (Thrombolysis in Myocardial Infarction) (36) and GRACE (Global Registry of Acute Coronary Events) (37) risk scores have been widely validated and are commonly used to estimate short- and long-term mortality in patients with acute coronary syndromes. These scores incorporate clinical, electrocardiographic, and laboratory variables to stratify risk, enabling clinicians to identify patients who may benefit from more intensive monitoring or early invasive strategies. However, these tools provide a static snapshot of risk and may not adequately reflect dynamic changes in a patient's clinical trajectory.

In real-world practice, patients often present with complex and evolving physiology. For example, a STEMI patient with initially stable hemodynamics may develop subtle signs of volume overload or low cardiac output within hours. Physical examination findings may lag behind pathophysiological deterioration, and reliance on static risk scores alone may lead to delayed recognition of clinical worsening. As such, dynamic, bedside assessment tools are increasingly being recognized as valuable adjuncts to traditional prognostic models.

## POCUS and acute heart failure in STEMI

The impact of acute heart failure on short- and long-term prognosis in patients admitted with STEMI is well established. In a seminal study conducted in the late 1960s, Drs. Killip and Kimball demonstrated that in-hospital mortality increased with the severity of heart failure, ranging from the absence of pulmonary rales at one end to the presence of cardiogenic shock at the other (Killip classification) (2). Decades later, the degree of acute heart failure remains one of the most important prognostic markers in myocardial infarction. More recent evidence shows that, beyond in-hospital outcomes, the Killip classification also predicts events up to five years after admission in patients with both ST-elevation and non–ST-elevation myocardial infarction (38).

Motivated by these findings and aware of the limitations of physical examination in assessing the degree of congestion, we proposed in 2020 a reclassification of the Killip system based on lung ultrasound findings, termed the LUCK (Lung Ultrasound Combined with Killip) classification (30). This novel approach demonstrated a significantly higher area under the curve to predict in-hospital mortality compared to the original Killip classification, with a net reclassification index of 18%. A key finding of this study was the negative predictive value of the LUCK classification for in-hospital mortality, which reached 98.1%. This highlights the higher sensitivity of lung ultrasound, enabling more accurate identification of patients who do not develop congestion in the context of STEMI—those who ultimately exhibit a better prognosis. Notably, 32% of patients initially classified as Killip class I (absence of pulmonary rales on auscultation) had at least one positive lung field on ultrasound.

Similarly, a Spanish research group developed a different score from a multicenter cohort, also based on the superior accuracy of lung ultrasound compared to physical examination. In this score, patients classified as Killip I with at least one positive lung field, as well as those classified as Killip II but without pulmonary congestion, were reclassified as Killip I pLUS (39). This new score not only demonstrated a higher area under the curve compared to the original Killip classification but also outperformed the LUCK classification in predicting in-hospital mortality and combined cardiovascular outcomes at one year. This finding was later confirmed in the cohort where LUCK was originally developed (40). Regarding medium- and long-term outcomes, data from the same multicenter cohort revealed that the presence of subclinical congestion (defined as at least one positive zone, comprising 14% of the sample) was associated with a fivefold increased risk of death or hospital readmission due to heart failure or acute coronary syndrome at 30 days (41), and a threefold increase in the combined endpoint at one year (42).

It is important to emphasize that while the presence of even a single positive zone on lung ultrasound in the context of STEMI carries significant prognostic value, it should not be formally interpreted as pulmonary congestion. As previously discussed, the diagnosis of pulmonary congestion requires at least two bilateral positive zones. Furthermore, even when pulmonary congestion is present, it may not be directly attributable to left ventricular systolic or diastolic dysfunction. Alternative mechanisms—such as increased pulmonary vascular permeability due to systemic inflammation or concomitant lung disease—may also produce B-lines. These potential confounders were not consistently excluded in some of the previously referenced studies.

## POCUS and cardiogenic shock in STEMI

Cardiogenic shock represents the most severe form of acute heart failure and remains a major cause of in-hospital mortality in patients with STEMI. It is characterized by inadequate tissue perfusion due to impaired cardiac output, often accompanied by hypotension, elevated filling pressures, and end-organ hypoperfusion. Despite advances in revascularization and mechanical circulatory support, mortality in CS continues to exceed 30%–40% in contemporary cohorts. As early recognition and protocol-based management are essential to improve survival, the Society for Cardiovascular Angiography and Interventions (SCAI) proposed a staging system emphasizing a dynamic and multidimensional approach to shock classification, based on clinical criteria to risk-stratify patients with CS from stages A to E (43).

Lung ultrasound may serve as a useful adjunct to the SCAI shock classification, particularly in stages A and B, where it enables detection of subclinical pulmonary congestion that may not be apparent on physical examination alone. In a previous study conducted by our group, higher SCAI shock stages were independently associated with an increased number of positive LUS zones, with an adjusted odds ratio of 2.2 (95% CI: 1.9–2.5; *P* < 0.001) (44). This suggests a 2.2-fold increase in the odds of detecting additional positive lung zones with each incremental stage in the SCAI classification.

In another study, we observed that left ventricular end-diastolic pressure (LVEDP), unlike B-lines on LUS, was not independently associated with in-hospital mortality (OR: 1.00; 95% CI: 0.97–1.03) or the occurrence of cardiogenic shock (OR: 1.01; 95% CI: 0.97–1.05) (45). Although some authors have proposed elevated LVEDP as part of the diagnostic criteria for cardiogenic shock—alongside clinical and hemodynamic parameters—pulmonary congestion identified by LUS appears to be a stronger marker. Furthermore, these findings call into question the hemodynamic rationale that increased left ventricular pressures—followed by elevated capillary pressure and pulmonary congestion in acute myocardial infarction—underlie the appearance of B-lines on point-of-care ultrasound (POCUS), raising the possibility that B-lines may instead be associated with other mechanisms such as inflammation.

Recently, our group proposed a novel method that incorporates LVOT-VTI into lung ultrasound assessment, as it offers key insights into global systolic function and cardiac output (46), similar to the Diamond Forrester classification (47) in this setting, in which although it utilizes precise cutoff points of Swan-Ganz measured pulmonary artery occlusion pressure (18 mmHg) and cardiac index (2.2 L/min/m^2^), it is both time-consuming and invasive. This non-invasive approach aims to detect subclinical abnormalities and identify, at an earlier stage, patients at risk of clinical deterioration despite the absence of overt signs on physical examination. The combination of these two ultrasound parameters forms the basis of the LUV classification (Lung Ultrasound and Velocity Time Integral), which categorizes patients into four distinct hemodynamic phenotypes according to the evidence of pulmonary congestion as defined by ≥3 positive zone scans and evidence of hypoperfusion as defined by a low VTI ≤ 14 cm. LUV classification A was defined as the absence of pulmonary congestion and normal LVOT-VTI; LUV classification B as the presence of pulmonary congestion and normal LVOT-VTI; LUV classification C as the absence of pulmonary congestion and low LVOT-VTI; and LUV classification D was defined as the presence of pulmonary congestion and low LVOT-VTI. This classification allows clinicians to move beyond simple binary distinctions (wet vs. dry, warm vs. cold) and into more nuanced bedside phenotyping. We have found in-hospital mortality rates of 0% for LUV A, 3% for LUV B, 12% for LUV C, and 45% for LUV D. The LUV classification demonstrated high predictive accuracy for in-hospital mortality (AUC = 0.915). Additionally, among patients not in Killip class IV at admission, the incidence of cardiogenic shock within 24 h increased progressively across LUV categories: 0% (A), 5% (B), 12.5% (C), and 30.8% (D), with an AUC of 0.90 for predicting shock. Moreover, the LUV classification outperformed LUCK and other LUS-based protocols in predicting in-hospital mortality and the development of cardiogenic shock within 24 h (48). This highlights the importance of hemodynamic assessment in the prognosis of such patients. While LUS plays an undeniable role in assessing STEMI patients, the incorporation of the LVOT-VTI variable provides an important hemodynamic assessment parameter that is more accurate than LUS alone. Acute hemodynamic dysfunction is undoubtedly a marker associated with poor prognosis in these patients. The incorporation of LUS with LVOT-VTI enhances the prognostic ability for adverse outcomes in patients without marked clinical signs of hemodynamic compromise. Notably, it can identify patients at risk of developing CS who might otherwise be classified as low-risk by other traditional methods.

For many years, survival rates in STEMI complicated by cardiogenic shock remained largely unchanged. During this period, primary percutaneous coronary intervention was the only intervention consistently associated with improved outcomes. However, in the late 2010s, the implementation of standardized management protocols with clearly defined thresholds for therapeutic escalation led to a significant improvement in survival—28% in comparison to historic controls—marking the first major advance in decades for this high-risk population (49). Among the thresholds used to guide escalation to mechanical circulatory support, cardiac power output (CPO) and pulmonary artery pulsatility index (PAPI) require invasive right heart catheterization, which is time-consuming and not always readily available. In this context, POCUS may serve as a valuable non-invasive alternative, particularly when rapid decision-making is critical to impact outcomes.

Of note, the absence of B-lines in a hypotensive STEMI patient should raise suspicion for an alternative etiology of shock, such as right ventricular infarction, pericardial tamponade, or massive pulmonary embolism. In these contexts, volume status may be low or normal, and pulmonary congestion may be minimal. Thus, LUS contributes not only to diagnosis but also to shock phenotype classification, which is increasingly recognized as central to guiding individualized therapy.

## Systematic POCUS evaluation

In a prospective cohort of 262 patients with acute coronary syndrome, systematic hand-held echocardiography demonstrated good-to-excellent agreement with comprehensive transthoracic echocardiography (TTE) across key parameters (Cohen's κ 0.60–1.00), achieving an overall negative predictive value of 95%. It was completed in a mean of 7.7 ± 1.6 min—about 5 h earlier than standard TTE—and identified clinically important cardiac abnormalities in 50% of cases, and altered management in 42%, with 85% of exams deemed sufficient to forego further imaging (50). A similar single-center study in patients hospitalized with acute myocardial infarction (*N* = 82) found that handheld echocardiography (using a V-scan) correlated well with standard TTE for global left ventricular function (concordance coefficient 0.75) and overall wall-motion assessment (0.69), though the agreement was weaker for regional wall-motion and structural abnormalities, supporting its role as a rapid adjunct rather than a replacement for comprehensive TTE (51).

**Figure 4:**
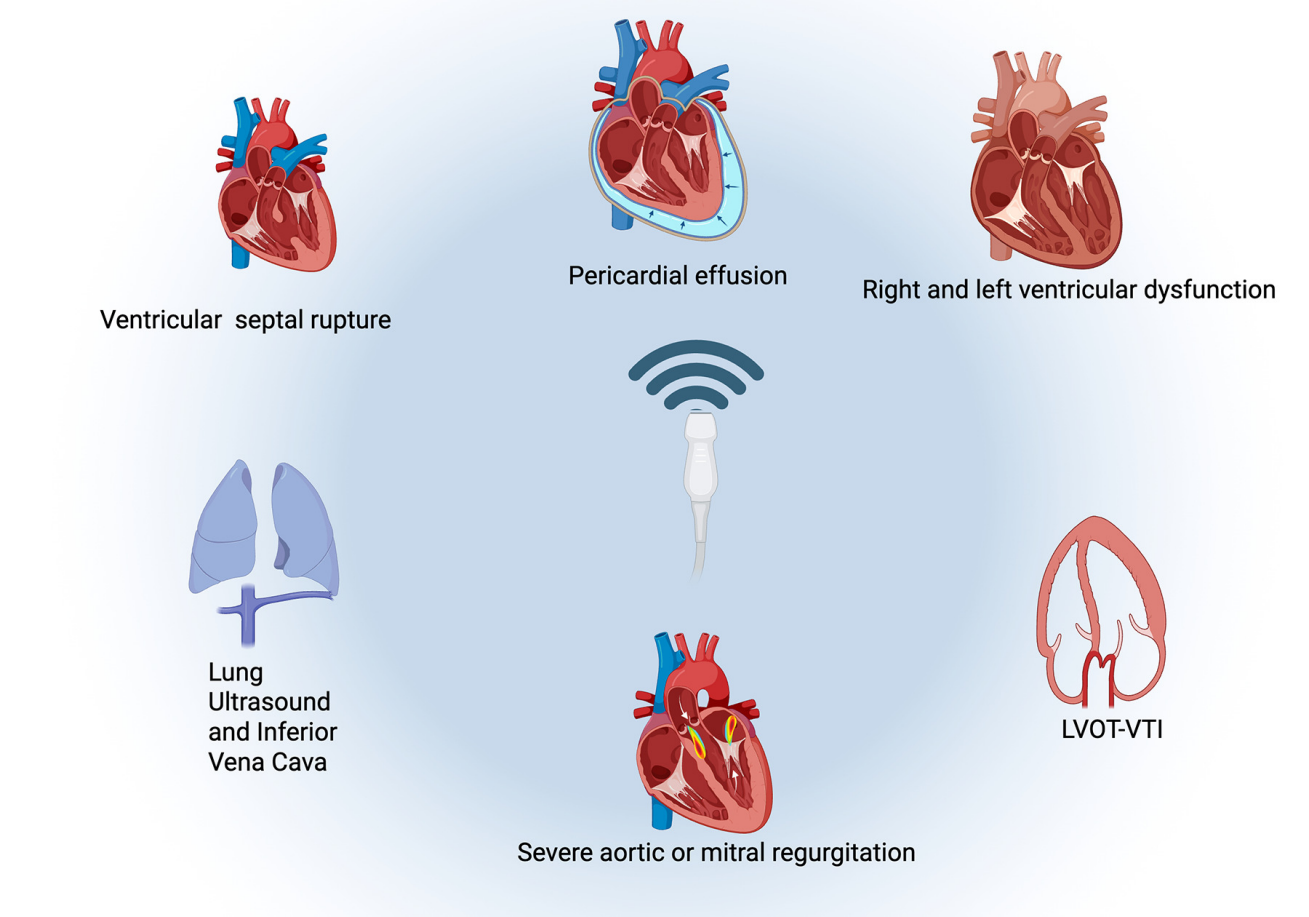
Checklist of parameters suggested in a systemic evaluation protocol. Figure created in Biorender.com.

Moreover, a systematic POCUS assessment such as the Focused Assessment in ST Elevation Myocardial Infarction (FASTEMI) is feasible and might change diagnosis and/or medical management in 12% of patients admitted with STEMI in the emergency room (52). The FASTEMI protocol involves performing an 8-zone LUS to assess for B-lines, screening for mechanical complications, identifying severe left-sided valvular regurgitations, assessing left and/or right ventricular dysfunction, measuring the LVOT-VTI, and estimating central venous pressure (CVP) through inferior vena cava (IVC) evaluation (Figure 4). POCUS enables rapid detection and facilitates early intervention, often before formal imaging can be arranged.

Although some authors have proposed using filling pressures, such as the E/e′ ratio, to assess critically ill patients (53), this approach can be time-consuming and is often unreliable in the setting of STEMI. As previously discussed, LVEDP demonstrated only a weak correlation with LUS findings and was not independently associated with in-hospital mortality or the development of CS (45). Pulmonary congestion in acute heart failure complicating STEMI represents a complex pathophysiological process that extends beyond simple fluid overload or elevated filling pressures.

In the context of STEMI, there are several clinical scenarios where the rapid hemodynamic evaluation provided by POCUS is particularly beneficial. Even patients in SCAI A or Killip Class I/II, POCUS can identify evolving congestion or low cardiac output before overt clinical deterioration. In cases of delayed hospital presentation (>24 h), POCUS enables detection of mechanical complications — such as papillary muscle rupture, ventricular septal defect, or pericardial tamponade—enabling life-saving interventions. Furthermore, in patients with complex presentations, including sepsis, pneumonia, or renal dysfunction, the use of POCUS supports tailored fluid and vasopressor strategies based on real-time cardiac and pulmonary findings. These scenarios exemplify the potential of POCUS to guide not only diagnosis and prognosis but also initial therapeutic decision-making in STEMI.

## Future perspectives

POCUS, particularly when integrated with LUS and LVOT-VTI, represents a paradigm shift in bedside hemodynamic assessment in STEMI. While current evidence supports the use of POCUS in early evaluation, several important directions are emerging that may further enhance its utility, accessibility, and impact.

### Integration into standardized STEMI pathways

Despite its diagnostic and prognostic value, POCUS is not yet routinely incorporated into formal STEMI management algorithms. Future practice guidelines may benefit from integrating focused ultrasound protocols into existing workflows—particularly for patients presenting with atypical symptoms, signs of heart failure, or suspected hemodynamic instability. Structured protocols, such as FASTEMI (52), combining lung ultrasound and LVOT-VTI could be used to enable early identification of high-risk phenotypes such as evolving cardiogenic shock, subclinical pulmonary edema, or mechanical complications.

### Artificial intelligence and automated interpretation

Technological innovations, particularly in artificial intelligence, are poised to reduce barriers to adoption by simplifying image acquisition and interpretation. AI-assisted POCUS platforms are already being developed to automatically detect B-lines, assess LV function, and even calculate VTI. These tools could make high-quality ultrasound assessments accessible to a broader range of clinicians—including non-cardiologists—and standardize reporting across institutions. Integration of AI into POCUS workflows may reduce operator dependency and training burdens while ensuring consistent, evidence-based decision support.

### Education, training, and credentialing

As the role of POCUS expands, formalized education and credentialing will become increasingly important. Interdisciplinary training programs, simulation-based learning, and online certification platforms can help standardize competence across emergency medicine, cardiology, and critical care. Establishing minimum standards for training and image interpretation—similar to advanced cardiac life support protocols—could improve the quality of POCUS implementation while maintaining patient safety.

### Research and clinical trials

Several studies have demonstrated the prognostic value of bedside sonographic parameters in risk stratification and prediction of adverse events (Table 1). However, despite reinforcing POCUS's potential as a prognostic marker, it remains unclear whether its application to guide therapeutic decisions (e.g., adjustments in hemodynamic support, selection of inotropic agents, or reperfusion strategies) translates into improved clinical outcomes such as reduced mortality or heart failure readmissions. Randomized clinical trials with standardized POCUS-guided intervention protocols are therefore needed to delineate the true impact of this approach on the prognosis of patients with acute myocardial infarction. Key research priorities include validating the LUV classification in diverse populations, evaluating the impact of early POCUS-guided therapy on mortality and heart failure rehospitalization, and determining cost-effectiveness compared to standard care.

**Table 1:** Studies of point-of-care ultrasound assessment in STEMI.

Study	*N*	Primary Outcome	Moment of assessment	AUC	Comparatives AUC
Araujo et al. (30) (LUCK)	215	In-hospital mortality	Pre pPCI	0.89	Killip: 0.86
Carreras-Mora et al. (39) (Killip pLUS)	373	In-hospital mortality	Within 24 h	0.90	Killip: 0.85 LUCK (30): 0.83
Parras et al. (54)	200	Heart failure	Pre pPCI	0.91	—
Araiza-Garaygordobil et al. (55)	226	Composite of death for any cause, new episode or worsening of heart failure, recurrent myocardial infarction, and cardiogenic shock at 30 days	Within 24 h	0.73	—
Machado et al. (46) (LUV)	308	In-hospital mortality	Within 24 h	0.915	Killip: 0.846
Machado et al. (48)^a^ (LUV)	145	In-hospital mortality	Pre pPCI	0.940	LUCK (30): 0.707 Killip pLUS (39): 0.691 Araiza-Garaygordobil (55): 0.704

^a^ Excluded patients in Killip 4.

AUC, area under the curve; pPCI, primary percutaneous coronary intervention.

While the prognostic value of POCUS in STEMI is increasingly supported by observational data, evidence of its impact on therapeutic strategies remains limited. However, studies in ambulatory heart failure populations—such as the LUS-HF trial (28)—have demonstrated that LUS-guided diuretic therapy significantly reduces rehospitalization and improves functional capacity. Whether such benefits extend to the acute STEMI population, particularly in cases of subclinical congestion or early hypoperfusion, remains an open question. Future randomized trials are needed to determine whether therapeutic strategies guided by real-time POCUS findings (e.g., adjustment of vasopressors, fluids, or reperfusion timing) translate into improved clinical outcomes.

### Current limitations

Despite its growing use, several factors may limit the optimal implementation of POCUS in the STEMI setting. Image quality and interpretation are operator-dependent and may vary significantly with training level. The performance of certain assessments, such as LVOT-VTI, requires adequate acoustic windows and technical skill, which may not always be available in emergency scenarios. Several factors may lead to inaccurate estimation of LVOT-VTI, particularly in critically ill patients. Moderate-to-severe aortic valve disease (stenosis or regurgitation), subvalvular flow acceleration (e.g., basal septal hypertrophy), or mechanical circulatory support devices can all significantly affect the measurement of stroke volume. In atrial fibrillation or tachyarrhythmias, beat-to-beat variation further reduces reliability. Moreover, malalignment of the Doppler beam or incorrect positioning of the sample volume may result in underestimation of flow. Crucially, any delay to door-to-balloon time must be avoided. Therefore, we emphasize that POCUS should complement clinical evaluation—not replace—and not delay reperfusion strategies.

## Conclusion

Point-of-care ultrasound has emerged as a valuable bedside tool in the early evaluation of STEMI patients. In a glimpse into the future, its broader integration into clinical pathways, and supported by evidence-based protocols, may redefine the standard of care in acute myocardial infarction. Beyond its role in the initial assessment, POCUS contributes significantly to prognostic stratification, aids in the identification of alternative diagnoses, and facilitates the early detection of mechanical complications. With the promise of earlier recognition, tailored interventions, and improved outcomes, POCUS is well-positioned to become an essential component of modern cardiovascular emergency medicine.
