# Correction: Point-of-care ultrasound in the evaluation of STEMI patients

**DOI:** 10.3389/fcvm.2025.1698664

**Published:** 2025-09-29

**Authors:** Marco Vugman Wainstein, Guilherme Pinheiro Machado, Guilherme Heiden Telo, Anderson Donelli da Silveira, Luiz Antônio Nasi, Gustavo Neves de Araujo

**Affiliations:** ^1^ Cardiology Department, Hospital de Clinicas de Porto Alegre, Porto Alegre, Brazil; ^2^ Cardiology Department, Hospital Unimed Grande Florianópolis, Florianópolis, Brazil

**Keywords:** STEMI, point-of-care ultrasound, lung ultrasound, LVOT-VTI, acute heart failure, cardiogenic shock

There was a mistake in Figure 1 as published. Figure 1 currently contain the FASTEMI logo, that overlap with material included in another manuscript from our group that is still under submission elsewhere. Our team is concerned that this overlap could potentially affect copyright considerations if that manuscript is accepted. The corrected Figure 1 appears below.

**Figure 1:**
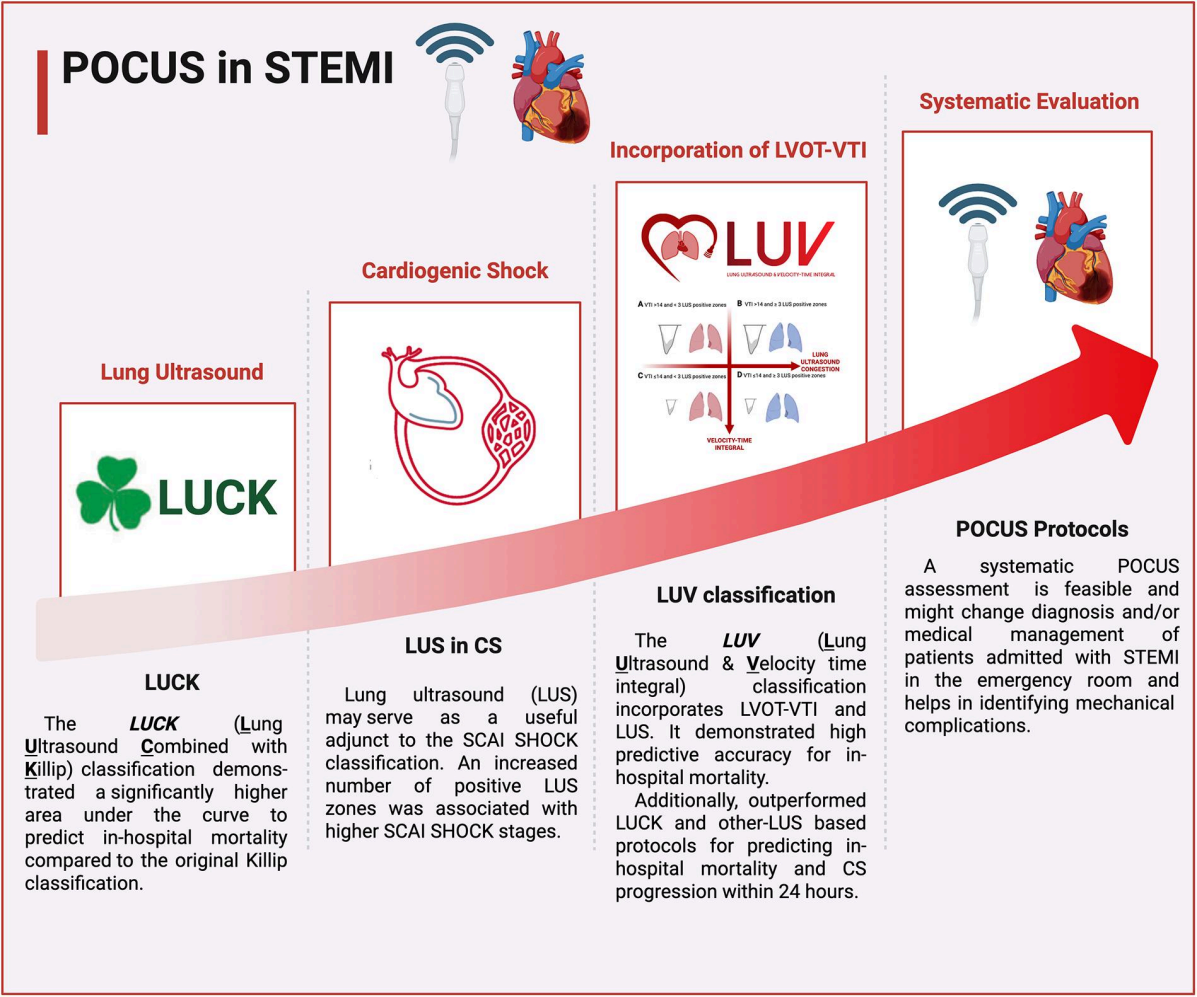


There was a mistake in Figure 4 as published. Figure 4 currently contain elements that overlap with material included in another manuscript from our group that is still under submission elsewhere. Our team is concerned that this overlap could potentially affect copyright considerations if that manuscript is accepted. The corrected Figure 4 appears below.

**Figure 4:**
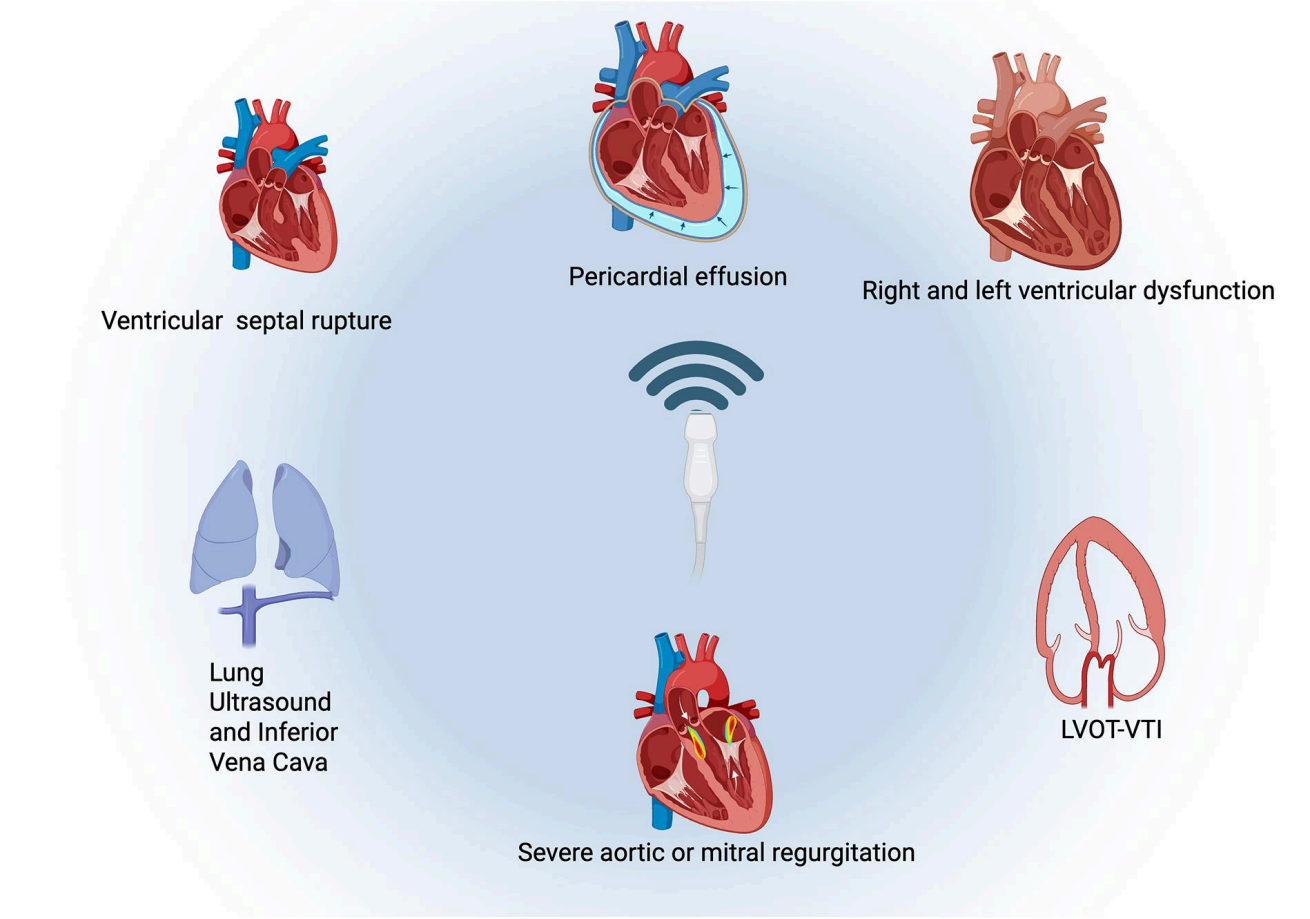


There was a mistake in the caption of Figure 4 as published. The corrected caption of Figure 4 appears below.

“Checklist of parameters suggested in a systemic evaluation protocol. Figure created in Biorender.com.”

The acknowledgements was omitted. The acknowledgements appears below.

**Acknowledgments**

The authors gratefully acknowledge the members of the research group for their essential contributions to this study. We would like to recognize, in alphabetical order: Andre Amon, Alan Pagnoncelli, Alexander G. Truesdell, Felipe C. Fuchs, Felipe P. Marques, Fernando Luis Scolari, Joao Pedro da Rosa Barbato, Luiz Carlos Corsetti Bergoli, Rafael Beltrame, Rodrigo Wainstein, Sandro Cadaval Goncalves, Tiago L. Leiria, and Wiliam Menegazzo.

The original version of this article has been updated.

